# Changes in brain activity with tominersen in early-manifest Huntington’s disease

**DOI:** 10.1093/braincomms/fcac149

**Published:** 2022-06-09

**Authors:** D J Hawellek, P Garces, A H Meghdadi, S Waninger, A Smith, M Manchester, S A Schobel, J F Hipp

**Affiliations:** Roche, Pharmaceutical Research and Early Development, Roche Innovation Center Basel, F. Hoffmann-La Roche Ltd., Basel 4070, Switzerland; Roche, Pharmaceutical Research and Early Development, Roche Innovation Center Basel, F. Hoffmann-La Roche Ltd., Basel 4070, Switzerland; Advanced Brain Monitoring Inc., Carlsbad, CA 92008, USA; Advanced Brain Monitoring Inc., Carlsbad, CA 92008, USA; Ionis Pharmaceuticals Inc., Carlsbad, CA 92010, USA; Roche, Pharmaceutical Research and Early Development, Roche Innovation Center Basel, F. Hoffmann-La Roche Ltd., Basel 4070, Switzerland; F. Hoffmann-La Roche Ltd, Basel 4070, Switzerland; Roche, Pharmaceutical Research and Early Development, Roche Innovation Center Basel, F. Hoffmann-La Roche Ltd., Basel 4070, Switzerland

**Keywords:** Huntington’s disease, EEG, neurodegeneration, biomarker

## Abstract

It is unknown whether alterations in EEG brain activity caused by Huntington’s disease may be responsive to huntingtin-lowering treatment. We analysed EEG recordings of 46 patients (mean age = 47.02 years; standard deviation = 10.19 years; 18 female) with early-manifest Stage 1 Huntington’s disease receiving the huntingtin-lowering antisense oligonucleotide tominersen for 4 months or receiving placebo as well as 39 healthy volunteers (mean age = 44.48 years; standard deviation = 12.94; 22 female) not receiving treatment. Patients on tominersen showed increased resting-state activity within a 4–8 Hz frequency range compared with patients receiving placebo (cluster-based permutation test, *P* < 0.05). The responsive frequency range overlapped with EEG activity that was strongly reduced in Huntington’s disease compared with healthy controls (cluster-based permutation test, *P* < 0.05). The underlying mechanisms of the observed treatment-related increase are unknown and may reflect neural plasticity as a consequence of the molecular pathways impacted by tominersen treatment.

Hawellek et al. report that patients with Huntington’s disease treated with the huntingtin-lowering antisense oligonucleotide tominersen exhibited increased EEG power in the theta/alpha frequency range. The underlying mechanisms of the observed changes are unknown and may reflect neural plasticity as a consequence of the molecular pathways impacted by tominersen treatment.

## Graphical Abstract

**Graphical abstract:**
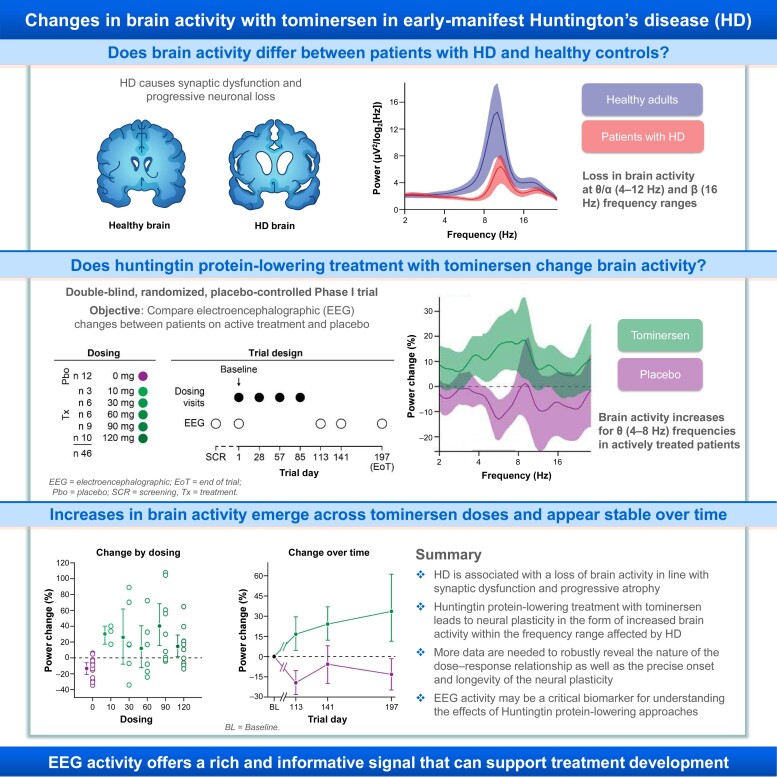

## Introduction

Huntington’s disease is a genetic, neurodegenerative, and ultimately fatal disease caused by a cytosine adenine guanine (CAG) trinucleotide repeat expansion in the huntingtin (*HTT*) gene, which results in the production of toxic mutant HTT protein (mHTT).^1–3^ Characterized by a triad of cognitive, behavioural, and motor symptoms, Huntington’s disease leads to functional decline and progressive loss of independence.^1,4^ The cause of the clinical decline in Huntington’s disease is a progressive neuronal loss that has a prominent early involvement of cell populations in the striatum, expanding towards cortical and system-wide atrophy in later stages of the disease.^5,6^

Synaptic dysfunction and loss^7,8^ and progressive neurodegeneration in Huntington’s disease may lead to changes in brain oscillatory activity as assessed with EEG.^9^ In particular, a decrease in synchronized neuronal activity with specific spectral patterns such as reductions in power in the classical alpha (α) band and at the theta-alpha (θ/α) border (7–8 Hz) has been reported in premanifest and early-manifest Huntington’s disease.^10–13^ These alterations suggest that EEG activities could serve as functional markers of disease progression. Importantly, EEG may also be a sensitive tool to monitor changes in brain function with HTT-lowering treatments that are currently in clinical development for Huntington’s disease.^14^

We analysed the resting-state EEG recordings of the Phase I/IIa study of tominersen (NCT02519036), an investigational antisense oligonucleotide (ASO) treatment for Huntington’s disease that lowers mutant and wild-type HTT in a non-allele selective fashion. In addition, we analysed EEG recordings from healthy controls (HCs). We assessed the EEG phenotype of Huntington’s disease as well as the changes in oscillatory activity after HTT-lowering treatment. We employed a data-driven approach that makes no assumptions on the specific frequencies or spatial location of potential effects. Our approach complements a set of previous analyses using a limited set of predefined EEG features^14^ and allowed for an unbiased mapping of Huntington’s disease and treatment-related effects.

## Materials and methods

### Clinical trial

Forty-six patients with Huntington’s disease participated in the randomized, double-blind, multiple-ascending-dose, placebo-controlled Phase I/IIa trial of tominersen (previously RG6042, HTT_RX_). Tominersen is an ASO complementary to a stretch of *HTT* mRNA and designed to induce its RNase H1-mediated degradation.

The trial was conducted in accordance with the Declaration of Helsinki. The trial protocol as well as detailed descriptions of bioanalytical and magnetic resonance imaging methods is available elsewhere.^14^ Briefly, the trial participants were between 26 and 65 years of age [mean = 47.02 years; standard deviation (SD) = 10.19 years; 18 female] with genetically confirmed Huntington’s disease (CAG repeat length ≥40) at an early-manifest Stage [Stage 1, defined as Unified Huntington’s Disease Rating Scale (UHDRS) Total Functional Capacity in the 11–13 range]. The trial extended across nine sites in Canada, Germany, and the United Kingdom.

### HC data

In addition, 39 healthy volunteers generally matched to the age and gender of the trial participants (mean 44.48 years; range 19*–*63; SD 12.94 years; 22 female) served as HC for the baseline visit EEG data. The HC data were recorded with the same set-up and procedures as compared with the clinical trial but was separate from the conduct of the clinical trial (ABM, Carlsbad, CA, USA).

### EEG acquisition

**Figure 1:**
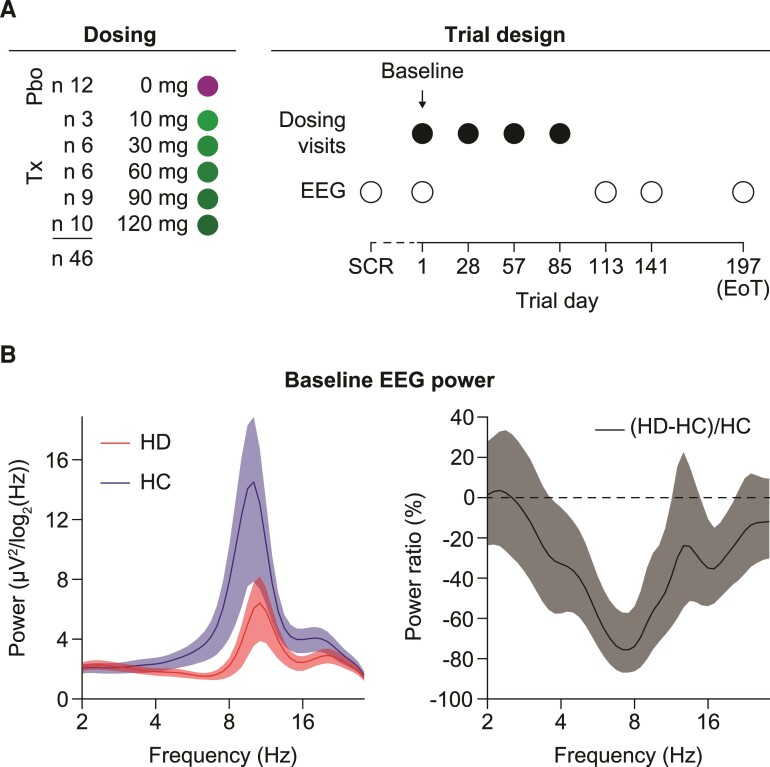
**Clinical trial design and the reduction of EEG activity in Huntington’s disease.** (**A**) Multiple-ascending-dose design of the tominersen Phase I/IIa trial. A total of 46 patients with Huntington’s disease was randomized to five different active dosing arms or placebo control. Resting-state EEG was recorded at SCR, baseline and Days 113, 141, 197 post-treatment (open circles) following the intrathecal injection of four monthly doses of tominersen (closed circles). (**B**) Left: Average EEG power spectra of patients with Huntington’s disease and matched HCs. We averaged the EEG activity across all 20 EEG electrodes for each participant. Right: Same data as on the left but shown as ratio between the group difference and the average level of power in the HC group. All error bars reflect a bootstrap estimate of the 95% confidence interval. EoT = end of trial, SCR = screening, Tx = treatment, Pbo = placebo.

Patients underwent EEG recordings at a screening visit 2–42 days prior to the baseline visit (mean = 24 days; SD = 10 days) (Fig. 1). Screening visit data were available for 44 of 46 patients.

Resting-state EEG was acquired for all patients with Huntington’s disease in two consecutive runs, each consisting of 5 min eyes open and 5 min eyes closed conditions (10 min total recording time for each condition). Two patients underwent only a single run of 5 min eyes open and 5 min eyes closed, and the HC data were recorded in a single run entirely. All data were recorded using the B-Alert® ×24 wireless EEG system by Advanced Brain Monitoring (Carlsbad, CA, USA) with 19 electrodes placed according to the International 10–20 system (Available EEG channels: Fz, F3, F4, Cz, C3, C4, P3, P4, Pz, O1, O2, T5, T3, F7, Fp1, Fp2, F8, T4, and T6—plus POz). Signals were recorded at a sampling rate of 256 Hz (linked mastoid reference) with a common mode rejection ratio of 105 dB and a high as well as a low pass filter at 0.1 and 100 Hz, respectively.

### Signal processing

All EEG data processing and analyses were done in MATLAB (2019a, version 9.6, Natick, Massachusetts: The MathWorks Inc.; 2010) using custom scripts and the Fieldtrip toolbox.^15^

We band pass filtered the EEG signals to within 1*–*30 Hz with a finite impulse response filter with an order of 512 (equivalent to 2 s). We rejected artefacts semi-automatically through a sequence of steps: (i) elimination of bad channels, (ii) rejection of periods where subjects were drowsy, as determined by visual inspection of change in EEG rhythms (fading of α oscillations and appearance of prominent low frequency oscillations), (iii) rejection of sections with large transient artefacts such as muscle bursts or movements, (iv) independent component analysis (FastICA)^16^ followed by identification and rejection of artefactual components (e.g. ocular, muscular, cardiac), (v) iteration of Points (iii) and (iv) if necessary, and (vi) interpolation of bad channels. After artefact rejection, we re-referenced the data to the common average. All analyses presented here are based on the eyes closed resting state recordings with a minimum of 217 s of clean data available across participants (range 234–574 s; HC 217–287 s).

We used Morlet Wavelets (0.33 octaves frequency resolution, f/σ_f_ = 10.24, window length of 5 σ_t_, 75% window overlap) to estimate power spectral densities and connectivity measures at 47 frequencies at f = 2^F^ Hz with the exponent F ranging from 1 (2 Hz) to 4.83 (28.4 Hz) in 0.083 steps. We also estimated the weighted phase lag index (wPLI) as well as orthogonalized amplitude correlations.^17,18^ The wPLI assesses connectivity at the high temporal resolution of signal phases, while the amplitude correlations assess slower co-fluctuations in power. Both metrics minimize the confounds of volume conduction onto the measured correlation between sites and can reveal structure in data that cannot directly be assessed by signal power alone.^18,19^ We quantified both connectivity measures for all available electrode pairs and then averaged the connectivity across all pairs at a given frequency for all patients for subsequent statistical analyses.

### Statistical analyses

**Figure 2:**
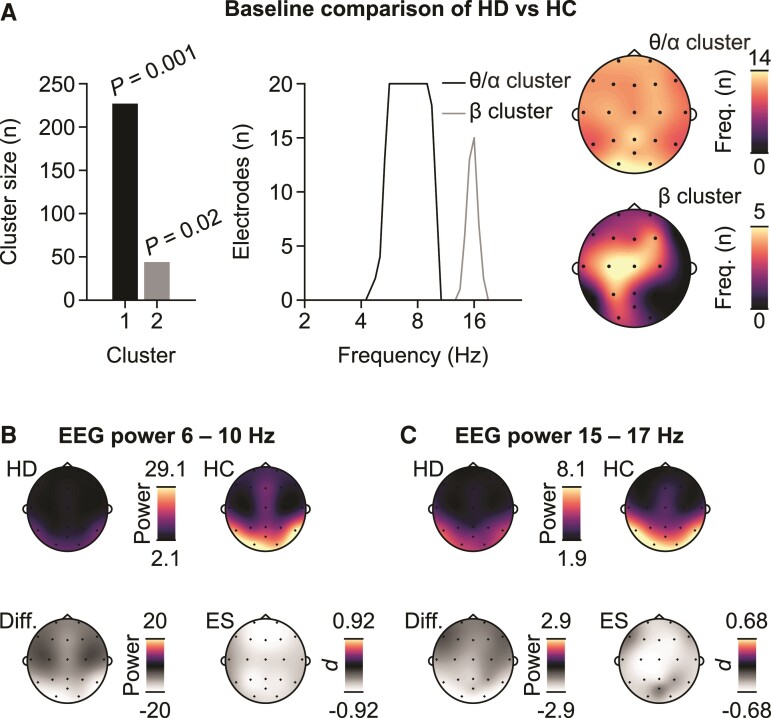
**Patterns of differences between patients with Huntington’s disease and HCs.** (**A**) Cluster sizes and associated *P*-values (cluster-based random permutation test) as well as projections of the clusters onto the frequency and electrode dimensions for the test of a difference between patients (*n* = 46) and HCs (*n* = 39). (**B**) Power topographies corresponding to the frequency range of the θ/α cluster for patients –Huntington’s disease and HC as well as the difference between the two groups expressed as raw power difference and Cohen’s d as an estimate of the ES. (**C**) Same as B for the β cluster.

**Figure 3:**
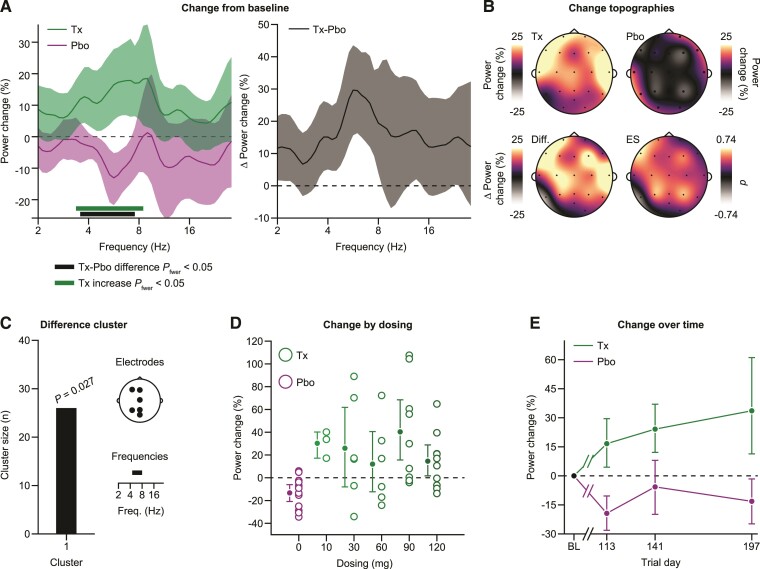
**Treatment with tominersen induces an increase in EEG activity.** (**A**) Left: comparison of the change from baseline in EEG activity between patients who received tominersen treatment (green) and patients who received placebo (purple). A significant cluster of neighbouring differences (*P* = 0.021, cluster-based permutation test, family-wise error rate corrected) between treated (*n* = 34) and placebo (*n* = 12) patients as well as a cluster of neighbouring frequencies with significantly increased EEG activity in the treatment cohort (*P* = 0.003, cluster-based permutation test, family-wise error rate corrected) are shown as a thick lines. Right: Same data are displayed as the difference in the change from baseline between treated and placebo patients. All error bars reflect bootstrap estimates of the 95% confidence interval. (**B**) Topographies of the change in power from baseline averaged across the frequencies with cohort differences shown separately for the treatment and placebo patients. The bottom row topographies display the difference between the cohorts and the associated Cohen’s d as an estimate of ES. (**C**) Cluster size, associated *P*-value (random permutation test) and contributing electrodes, and frequencies for the difference cluster between treatment and placebo patients when data are not averaged across electrodes for each patient but the analysis is performed in the full frequency by electrode space. (**D**) Change of EEG activity from baseline by dosing levels. **E)** Change of EEG activity from baseline separately for the three post-treatment visits. For (**D**) and (**E**), we averaged the change in power from baseline within the significant cluster of differences between treatment and placebo patients, detected when performing the analysis in the full electrode-by-frequencies space (**C**). Pbo = placebo, Tx = treatment.

We tested for differences in EEG activity between groups of participants using cluster-based permutation tests.^20^ Briefly, we performed Wilcoxon rank-sum tests as a first-level statistic. We considered a cluster to be the contiguous extend of tests that exceeded a significance threshold in the frequency *(n* = 47)×electrode (*n* = 20) space with a total of 940 comparisons. We used *P* < 10^−2.3^ (=0.005) as cluster-forming threshold for the Huntington’s disease to HC comparison and *P* < 10^−2^ for the treatment to placebo comparisons. Variations in the cluster-forming thresholds led to highly similar results. We used the spatial layout of the EEG electrodes to consider neighbouring electrodes as electrode pairs that were directly physically adjacent. We estimated the overall significance of the resulting cluster sizes by comparing the results to an empirical null-hypothesis distribution obtained by randomly permuting the group labels of the participants (5000 permutations). Importantly, comparing the actual cluster sizes to the distribution of maximal cluster sizes under permutation addressed the multiplicity of comparisons by controlling the family-wise error rate across the entire frequency-by-electrode space.^21^ To visualize the shape and extent of the emerging clusters we summed the number of significant cluster bins for a given frequency (e.g. Fig. 2A, maximum of 20) or electrode (maximum of 47). We used the electrodes and frequencies belonging to a group-level cluster as mask to average the EEG activity of individual participants for subsequent analyses such as cross-sectional associations (e.g. Fig. 2D). For the comparison of treatment and placebo cohorts, we also employed cluster-based permutation statistics for power spectra averaged across all electrodes (Fig. 3B). We applied the same procedures as described above and considered neighbouring frequency bins with rank-sum tests exceeding a significant threshold to form the clusters.

Throughout all results, bivariate correlations denoted with *r* reflect the Pearson correlation between the variables while *ρ* reflects the Spearman rank correlation for the same variable pair.

We assessed the performance of a multivariate decoder trained to classify participants as patient or HC based on baseline EEG activity using a linear discriminant analysis (LDA). We used leave-one-out cross-validation and assessed the decoders performance using the area under the curve (AUC) of the resulting receiver operating characteristic as well as the confusion matrix. The confusion matrix shows the fractions of correctly and incorrectly labelled participants for each possible category of correct and false classification.

**Figure 4:**
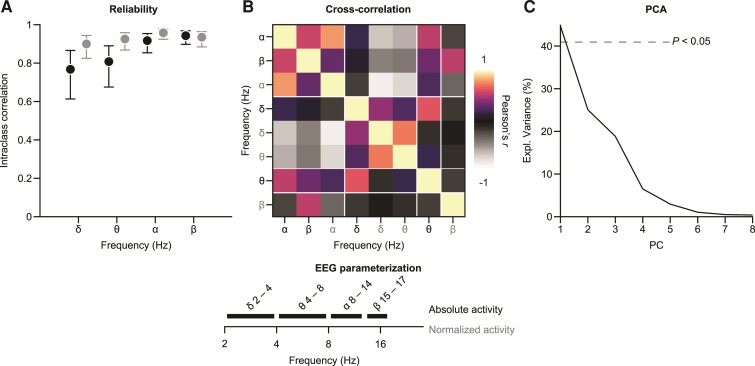
**Parametrizing EEG signal power in Huntington’s disease.** We investigated the reliability and interdependence of eight EEG features derived from four frequency ranges (δ, θ, α, β) for absolute and normalized EEG activity. (**A**) Intraclass correlation of the EEG parameters between screening and baseline visits of the clinical trial (*n* = 44). Error bars reflect 95% confidence intervals. (**B**) Pairwise cross-sectional correlations between all EEG features at baseline (*n* = 44). Features were sorted according to their optimal modularity indicated by white lines. (**C**) Explained variance of PC of a PCA across the EEG parameters. We show the 95th percentile of the amount of variance explained by random projections of the data with Gaussian weights as a dotted line for reference. PC = principal components, PCA = principal component analysis.

Using data from the screening visit and the baseline visit, the intraclass correlation coefficient (ICC) was assessed for EEG-derived parameters on short term, within-patient intervention-free follow-up. We used the Type 1–1 ICC^22^ as a measure of the parameters test-retest reliability (Fig. 4A). The ICC assesses reliability by comparing between and within-subject variability through a one-way random effects model. The ICC is close to 0 when there is no reliability and it approaches 1 when there is a perfect agreement between all measurements. We used the function modularity_und of the brain connectivity toolbox^23^ for visually grouping the correlation matrix of the EEG signal power parameters (Fig. 4B).

### Data availability

The data are not publicly available due to the confidentiality requirements of the clinical trial data and the protection of the privacy of the patients.

## Results

### EEG activity differs between patients with Huntington’s disease and HCs

Patient baseline demographics and characteristics have been described previously.^14^ We first investigated differences in EEG activity between the early-manifest Huntington’s disease cohort (Stage 1, defined as UHDRS Total Functional Capacity in the 11–13 range) of the Phase I/IIa trial (*n* = 46, Fig. 1A) and HCs matched in age and gender (*n* = 33). Visual inspection showed a marked loss in power in the 4–12 Hz range as well as within the beta (β) band at approximately 16 Hz (Fig. 1B). The peak difference in EEG activity was slightly lower (about 8 Hz) than the α activity peak, the most prominent part of the power spectrum in both HC and Huntington’s disease at about 8–12 Hz.^10–13^ The activity reduction in EEG activity reached an average of about 70% at 7–8 Hz.

To statistically assess the patterns of differences in EEG activity in a data-driven way, we used cluster-based permutation statistics across all electrodes and frequencies. In line with the qualitative observations above, we found two significant clusters (*P* < 0.05, permutation tests, family-wise error-controlled) in the low θ/α and beta (β) frequency ranges (Fig. 2A).

The scalp topographies of the two clusters suggested that the group differences in brain activity were widespread across the scalp. All electrodes contributed to the θ/α cluster. The β cluster was more localized to central portions of the scalp (Fig. 2A).

We next investigated how the cluster topographies relate to the underlying scalp distribution of signal power. To this end, we averaged the power corresponding to the key frequencies of the statistical θ/α and β clusters, i.e. 6–10 Hz as well as 15–17 Hz for each participant, respectively (Fig. 2B and C). In Huntington’s disease and HC, the θ/α activity had a posterior to anterior gradient. The gradient pattern was reduced in Huntington’s disease as compared with HC in a relatively homogeneous way, leading to a similar effect size (ES) of the group comparison across the scalp. The scalp distribution of β activity followed a similar posterior to anterior pattern and more homogenous pattern of the ES of the group comparison. These observations suggest that the EEG activity reductions in patients with Huntington’s disease reflected preserved spatial patterns of EEG activity that were reduced globally as compared with HC.

The differences between Huntington’s disease and HC suggest that multivariate analyses based on the EEG may robustly discriminate Huntington’s disease patients from HCs. We therefore performed multivariate decoding using a cross-validated LDA to distinguish Huntington’s disease from HC, using the average EEG power spectrum per patient as features. The patients with Huntington’s disease were sensitively and specifically discriminated with an AUC of a receiver-operating characteristic of AUC = 0.8 (Supplementary Fig. 1A).

A caveat of the analyses presented above is that the HC EEG data have been acquired in experiments that were separate from the clinical trial. However, the use of the same recording devices and highly similar procedures along with the results above that are well in line with literature reports on EEG alterations in Huntington’s disease, give us confidence in the observed differences.

In order to further explore the HD related EEG phenotype we used the same approach to test for a difference between HD and HC for other EEG resting state parameters (Supplementary Fig. 2). In sum, we found two components of altered levels of brain activity in patients with Huntington’s disease allowing for a sensitive discrimination of patients from HC based on resting EEG.

### Associations of the baseline EEG alterations with Huntington’s disease progression

We next addressed whether the EEG activities in Huntington’s disease that were different from HC were associated with the patients’ baseline clinical status.

Important objective parameters informing about the disease progression in Huntington’s disease are a patient’s age, the number of CAG repeats in their *HTT* gene, and the disease burden score [CAG-age product (CAP)] integrating age and CAG. We used these three disease-relevant parameters to construct a linear model that significantly predicted the variability in EEG activity at baseline for the θ/α activity (*P* = 0.022, *r*^2^ = 0.2, general linear model) and not for the β activity (*P* = 0.063, *r*^2^ = 0.15, general linear model). We inspected scatter plots to better understand the relationship between the parameters and their relationship to the EEG activity (Supplementary Fig. 1B–D).

We next expanded the significant model for θ/α activity based on age, CAG, and CAP scores with additional parameters. The additional parameters reflected the clinical disease state and were the composite UHDRS (cUHDRS) at baseline,^24^ baseline levels of the cerebrospinal fluid (CSF) protein markers of neurodegeneration mHTT, and neurofilament light,^25^ as well as the baseline volume of the lateral ventricles.^14^ We performed nested model testing to assess whether the inclusion of the individual parameters would lead to a significant improvement over the reference model. We found that none of the additional parameters led to model improvements (all *P* > 0.05, likelihood-ratio tests).

Taken together, the data were broadly consistent with the view that the EEG activity of Huntington’s disease patients at baseline may reflect downstream consequences of the altered *HTT* gene driving the disease.

### Tominersen treatment changes EEG activity in patients with Huntington’s disease

We next investigated the effects of tominersen treatment on EEG activity. We averaged the EEG power spectra across all three post-treatment visits for each patient and pooled all patients on active treatment into one cohort. We then compared the longitudinal change from baseline between the treatment and placebo cohorts for the whole spectrum.

The longitudinal change from baseline revealed a significant enhancement of power in treatment compared with placebo (*P* < 0.05, permutation tests, family-wise error-controlled) (Fig. 3A). We next tested for each cohort separately whether there was also a significant change from baseline. A cluster of neighbouring frequencies that overlapped with the treatment–placebo differences exhibited significantly increased power as compared with baseline for the treatment group (*P* < 0.05, permutation tests, family-wise error-controlled). We found no significant change from baseline in a controlled test for the placebo cohort (*P* < 0.05, permutation tests, family-wise error-controlled). In sum, tominersen treatment led to increases in 4–8 Hz EEG activity that constituted a difference to the placebo cohort, who did not exhibit significant longitudinal EEG changes.

We next revealed the scalp topographies of the longitudinal change for the cohorts (Fig. 3B). The increases in EEG activity in the treatment cohort were widespread across the scalp and prominent in anterior portions extending centrally towards the posterior. The scalp topography of the placebo cohort had a homogenous appearance, leading to a difference between the groups that was not highly localized on the scalp but extending bilaterally from anterior to posterior portions of the electrode system. Thus, the increases in EEG activity due to tominersen treatment were present across the scalp. Of note, the differences between treatment and placebo cohorts shared similarities with the differences between Huntington’s disease patients and HCs in their dominating frequency range and widespread scalp distributions but were in opposite directions.

The above analyses only addressed the comparison of spectra averaged across all electrodes for each patient. In line with our previous analyses on the Huntington’s disease–HC differences, we next used cluster-based permutation statistics across the entire frequency-by-electrode space. A significant cluster between treatment and placebo cohorts also emerged in this wider search space, further confirming the robust difference between treatment and placebo that did not depend on the specific way the data were averaged across electrodes (*P* < 0.05, permutation tests, family-wise error-controlled). The contributing electrodes extended centrally across the scalp and the contributing frequencies fell within the 4–8 Hz range (Fig. 3C).

We next investigated if the increases in EEG activity depended on the dose of tominersen, as well as how the increases unfolded across the three post-treatment visits in time. To address these questions, we averaged the longitudinal EEG activity change for each patient using the difference cluster between treatment and placebo as an electrode–frequency mask (Fig. 3C). In other words, we extracted the EEG activity for each patient from those electrodes and frequencies that most strongly differentiated between the two cohorts.

We did not observe a systematic relationship to tominersen dose in the data (Fig. 3D). The treatment subgroup exhibited a considerable variability in the longitudinal change of the EEG and increases in activity were visible for patients of all dosing groups. We found no significant main effect of tominersen dose within the treatment group (*P* > 0.05, one-way analysis of variance with the factor dose with five levels corresponding to the active dosing groups 10–120 mg). Consistent with this observation, we also found no association between the ASO trough concentrations averaged across the CSF measurements for each patient who received treatment and their EEG activity change (Pearson’s *r* = –0.06, *P* = 0.72; Spearman *ρ* = –0.06, *P* = 0.7).

The increases in EEG activity due to tominersen treatment were detectable throughout the post-treatment observations (Fig. 3E). We tested whether the EEG activity increases exhibited consistent changes in time. We assessed whether the slope of a linear regression had a significant magnitude that would indicate a consistent linear change over time. We found no significant linear slopes (*P* > 0.05, permutation test).

The early-phase trial design with a limited number of patients did not allow for robustly assessing the clinical meaningfulness of the observed treatment-related increases in EEG activity. We observed a pattern of putative associations between the EEG activity increase and changes in clinical variables that was not indicative of adverse effects (Supplementary Fig. 3).

During the original clinical trial, several predefined EEG endpoints were included for which no consistent effects of treatment were found.^14^ We next assessed whether treatment effects would emerge for the predefined EEG endpoints when using the same procedures of pooling of data across visits and dose arms as above. These predefined EEG endpoints were selected based on the literature on resting-state EEG in Huntington’s disease: absolute signal power in the α (8–12 Hz), θ/α border (7–8 Hz) as well as δ (2–4 Hz) range, along with spatial gradients in the α (8–12 Hz) and δ (2–4 Hz) ranges and mean frequency across the (2–30 Hz) range.^10–13,26^ We averaged the predefined endpoints across post-treatment visits and pooled all patients on active treatment for a comparison with the placebo group. A pattern emerged that was very consistent with the results obtained with the cluster-based approach presented above. We found a median increase in activity for patients on active treatment of 14.4% compared with placebo for the θ/α border power change (*P* = 0.014, Wilcoxon rank-sum test). None of the other endpoints showed significant changes between the cohorts (all *P* > 0.05, Wilcoxon rank-sum test, median differences between active treatment and placebo groups: δ-power +5.7%, α-power +22.7%, mean frequency −0.08 Hz, δ-gradient +0.02, α-gradient −0.4). The overall pattern across predefined endpoints is in line with the idea that EEG activity increases across the 4–8 Hz range (Fig. 3A).

### Parametrizing EEG signal power for clinical trials in Huntington’s disease

Based on our analyses, four potentially informative spectral ranges of EEG signal power emerged –δ, θ, α and β. Power at each of these frequency bands could potentially serve as biomarker endpoints in Huntington’s disease clinical trials. In order to support more informed choices for parametrizing EEG signal power in future trials based on our data, we quantified the reliability (Type 1–1 ICC) as well as the interdependence of signal power across the different spectral components using absolute as well as normalized versions (Fig. 4).

The availability of short-term follow-up measurements before the start of tominersen treatment at screening and Day 1 visits (Fig. 1A) allowed for an intraclass correlation analysis of the spectral components for Huntington’s disease patients. We observed at least good-to-excellent reliabilities (ICC > 0.75) of all features (Fig. 4A). We observed a tendency for the ICC values to increase with frequency. Overall, the high ICC values indicate a high robustness of the EEG signal power features.

To assess the interdependence of the different spectral features, we computed a cross-correlation matrix of all features measured at baseline (Fig. 4B). The emerging pattern indicated that the features were strongly interdependent and dominated by two larger anti-correlated modules corresponding largely to the higher (α, β) and lower (δ, θ) parts of the spectrum. Interestingly, the absolute θ power that we found to be responsive to tominersen treatment in the previous analyses did not strongly follow the same correlation pattern, suggesting the 4–8 Hz part of the spectrum to be a signal with partially distinct and complementary information to the larger two modules of higher and lower EEG activity. In line with the observed correlation pattern among the spectral features, we observed the first principal component of the features to explain more than 40% of all variance across the patients (Fig. 4C).

In sum, we observed high intraclass correlations of the EEG signal power features suggesting that they represent suitable biomarker signals in Huntington’s disease trials. The correlations among features suggest some degree of redundancy across the spectrum, with an anti-correlation between the faster and slower components. Interestingly, the treatment-responsive part of the spectrum (absolute power in 4–8 Hz) emerged as a less redundant feature compared with others and may be of particular interest for future research into the utility of EEG-based biomarker read outs in Huntington’s disease.

## Discussion

We found baseline alterations in EEG activity of patients with Huntington’s disease consistent with literature reports^9^ that were partially counteracted by an increase in EEG activity after treatment with tominersen, an HTT-targeting ASO. Our observations suggest neural plasticity under tominersen treatment, the underlying mechanisms of which await further investigation.

At the time of writing this manuscript, the dosing in the Phase III trial of tominersen [GENERATION HD1 (NCT03761849)] has been halted as recommended by the trial’s independent monitoring committee. It is important to note that the data presented here stem exclusively from the earlier Phase (I/IIa) of clinical investigation with a fundamentally different dosing paradigm to GENERATION HD1. Since EEG recordings are not available from the GENERATION HD1 trial, future investigations will be needed to assess in detail the relationship between the EEG activity changes, drug exposure and clinical outcomes in the context of an HTT-lowering approach in Huntington’s disease.

Widespread changes in brain activity as measured by EEG are a prominent signature of neurodegenerative disorders.^9,27–29^ In particular, a slowing of brain activity with a reduction of faster activity and an increase in slow activity is a pattern often observed in the context of accumulating neurological damage. The reasons for such a converging pattern of global EEG changes in neurodegeneration are unclear. EEG activities in specific frequency ranges may reflect neurophysiological signatures of the information flow in brain circuits. For example, α activity has been linked to feedback processing in deep cortical layers,^30,31^ and slower activity such as θ activity may often reflect a coordination of cortical activity with subcortical brain structures, e.g. the hippocampus.^32^ However, a one-to-one relationship between activity at specific frequency ranges and brain function does not exist since several different neurophysiological processes may share overlapping frequency characteristics.

The progressive degeneration of cortico-striatal circuits and synaptic dysfunction and loss^7,8^ in Huntington’s disease may induce changes to frequency-specific activities through several mechanisms. EEG activity may change as a direct consequence of the loss of contributing synapses and circuits. That is, the loss of EEG activity could directly reflect the loss of certain cell populations. Alternatively, the changes may also reflect an indirect impact of Huntington’s disease on circuit activity for example due to compensatory neural plasticity. That is, the change in EEG activity could also result from the reorganization of specific cell populations. In both scenarios, the changes in brain activity due to Huntington’s disease reflect the functional consequences of an altered neuronal architecture. Along the same lines, the treatment-induced increase of EEG activity may reflect the impact of several different mechanisms, including the *de novo* gain of contributing synapses or circuits as well as plastic changes and reorganization of activity across existing circuits. Our observations highlight the opportunity for novel mechanistic investigations into the circuits level effects of HTT-lowering therapies in preclinical models^33^ and suggest that EEG activities may be an important biomarker in future Huntington’s disease trials for the advancement of non-allele-selective HTT-lowering therapies.

Tominersen treatment reduces levels of mHTT expression in patients with the goal to limit the consequences of the mHTT toxicity.^14^ HTT is a ubiquitously expressed protein with complex and unclear function.^3^ Importantly, HTT has no known direct role in mediating electrophysiological activity as compared with, for example, ion channels, for which pharmacodynamics effects often include direct electrophysiological signatures with a fast onset and transient duration during pharmacological interventions.^34–36^ The changes in brain activity with tominersen that unfold and remain stable over the time course of months likely reflect a sustained plastic change at the circuit level, downstream to a molecular cascade of HTT lowering which occurs under tominersen treatment.

The relatively low number of patients included in the trial limits any firm conclusions about the clinical impact of the observed changes. Replication in larger cohorts of patients will be necessary before the full potential of the EEG activities as biomarkers in Huntington’s disease can be assessed robustly.

The results presented here contrast the previous analysis of a set of predefined features based on the same EEG recordings that have not revealed consistent patterns of longitudinal change.^14^ Several important differences with the analyses presented here are worth noting. First, our data-driven approach was indiscriminant towards a particular scalp location or frequency range for detecting effects. The EEG activity found to be responsive to tominersen treatment (4–8 Hz) emerged at a frequency range mostly outside of the scope of the predefined features. Second, we employed a hierarchical testing approach by pooling across dosing groups and post-treatment visits for detecting an effect in a first step more sensitively. We then investigated the dependency of the detected EEG activity change on other factors (dose, time) in subsequent analyses. The data pooling may have enabled sufficient statistical power to detect changes in the treated patient cohort that remain undetectable when separating a limited number of patients into even smaller subgroups. Upon reanalysis of the predefined endpoint data used in the analyses of the clinical trial, a consistent pattern emerged with increased EEG activity for patients on active treatment at the θ/α border (7–8 Hz), a predefined EEG feature that partially overlapped with the effect detected here. In sum, the EEG offers a complex signal that can be parameterized and used in drug development in several different ways. Our findings underline the importance of data-driven approaches that can complement predefined EEG features to leverage the full potential of EEG as a functional biomarker in Huntington’s disease.

## Supplementary Material

fcac149_Supplementary_Data

## Abbreviations

α =: alpha
ASO =: antisense oligonucleotide
AUC =: area under the curve
θ/α =: theta/alpha
CAG =: cytosine adenine guanine
CAP =: CAG-age product
CSF =: cerebrospinal fluid
cUHDRS =: composite UHDRS
ES =: effect size
HC =: healthy controls
*HTT* =: huntingtin gene
HTT =: huntingtin protein
ICC =: intraclass correlation coefficient
LDA =: linear discriminant analysis
mHTT =: mutant HTT protein
SD =: standard deviation
UHDRS =: Unified Huntington's Disease Rating Scale
wPLI =: weighted phase lag index
