# Supplementary material for: Changes in brain activity with tominersen in early-manifest Huntington’s disease
Source: Brain Commun. 2022 Jun 9;4(3):fcac149. doi: 10.1093/braincomms/fcac149 (PMC9237739; doi:10.1093/braincomms/fcac149)
Supplement: fcac149_Supplementary_Data [file fcac149_supplementary_data.pdf]

Supplementary materials

Changes in brain activity with tominersen in early-manifest Huntington's disease

David J. Hawellek, Pilar Garces , Amir H. Meghdadi, Shani Waninger, Anne Smith, Marianne Manchester, Scott A. Schobel, Joerg F. Hipp

Differences between Huntington's disease and healthy controls (HC) for normalized electroencephalographic (EEG) activity and connectivity

We repeated the analyses comparing Huntington's disease and HC (**Fig. 2**) for additional EEG-derived measures (**Supplementary Figure 2**). Previous work has emphasized the importance of normalized EEG activity as well as measures of connectivity that can provide information about features of brain activity not detectable by absolute signal power alone.[1,2]

Normalizing the EEG spectra for each patient with the integral of power across all frequencies may suppress cross-sectional variability in the data that is not related to Huntington's disease but instead to other inter-individual differences. As a result, normalized EEG activity could principally be more sensitive to disease-specific factors. A consequence of the normalization is that differences between Huntington's disease and HC less clearly localize to specific frequencies within the spectrum. For example, a strong group-wise reduction in absolute EEG activity in one frequency range (e.g. alpha [α]) may trivially induce apparent increases in EEG activity at other frequency ranges through normalization. In other words, differences in normalized EEG activity relate more strongly to differences in the overall shape of the power spectra.

When comparing the normalized EEG activity between Huntington's disease and HC we observed a similar reduction in activity at about 8 Hz. In addition, the normalized spectra of patients with Huntington's disease was increased towards frequencies below 4 Hz (**Supplementary Figure 2A**), in line with the considerations about spectral shape above. Correspondingly, the cluster-based permutation tests revealed two clusters of differences (P <

0.05, permutation tests, family-wise error-controlled) of normalized EEG activity that we refer to as normalized α (α_N) and normalized delta (δ_N) clusters, respectively (**Supplementary Figure 2B**). The scalp topography for the δ_N cluster had an anterior to posterior gradient, while the α_N cluster was again widespread across the scalp.

We next applied the same steps of analysis to two measures of connectivity. The weighted phase lag index (wPLI) relies on the fast synchronization of oscillatory phases[3] while amplitude correlations rely on the slower co-fluctuation of signal amplitudes between electrodes.[1] For statistical analysis, we quantified the connectivity between all available electrode pairs for each participant at each frequency and averaged the connectivity across all electrode pairs for the comparison between Huntington's disease and HC.

The connectivity differences between Huntington's disease and HC dissociated from the patterns of signal power (**Supplementary Figure 2C, E**). Interestingly, both measures of connectivity did not reveal differences in the α range, where the signal power differentiated most strongly between Huntington's disease and HC.

For the wPLI, we found a cluster of increased connectivity in the δ_c range < 4 Hz (**Supplementary Figure 2C**, $P < 0.05$, permutation tests, family-wise error-controlled). The scalp topography of the cluster had a mostly peripheral distribution.

The amplitude correlations showed no differences between Huntington's disease and HC throughout the spectrum ($P > 0.31$, permutation tests, family-wise error-controlled).

Overall, we found robust changes in EEG activity in the patients with Huntington's disease. The low α range normalized power most strongly differentiated Huntington's disease from HC, while

the δ range was also informative for normalized EEG power and electrode connectivity as measured with the wPLI.

Supplementary figures

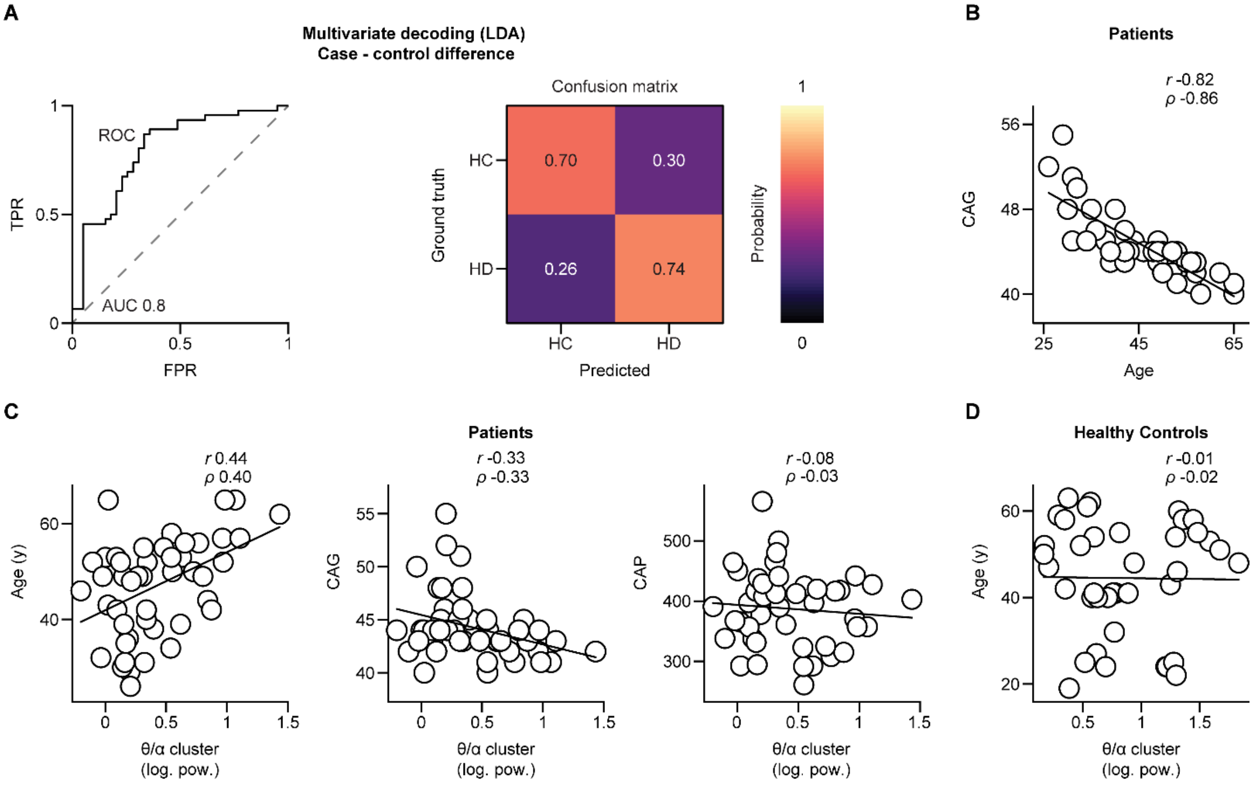

Supplementary Figure 1 Multivariate decoding of patients and HCs based on EEG and scatter plots for baseline clinical and EEG variables. A) Leave-one-out cross-validated performance of a linear discriminant, decoding patients and HCs based on the average spectra for each participant across all electrodes (**Fig. 1B**). Performance is shown in terms of the ROC, TPR, FPR and the confusion matrix. **B)** By recruiting patients with comparable disease progression, the patients who participated in the clinical trial exhibited a strong negative correlation between their age and the number of CAG repeats of the *mHTT* with younger patients exhibiting a higher

CAG repeat number (Pearson's $r = 0.82$, Spearman's $\rho = 0.86$, $P < 0.05$) **C)** To better understand the relationship between the reference model used in the main text that was composed of age, CAG and CAG-age product and the θ/α EEG activity, we additionally inspected scatter plots between the individual parameters. We observed that age (Pearson's $r = 0.44$, Spearman's rho = 0.4, $P < 0.05$) and the number of CAG repeats in the *HTT* (Pearson's $r = –0.33$, Spearman's $\rho =$ 0.33, $P < 0.05$) were among the strongest associations for the θ/α activity in the Huntington's disease cohort. Interestingly, age and CAG repeats had different signs in their associations, i.e. younger age and higher numbers of CAG repeats were associated with lower EEG activity. This pattern could have predominantly been driven by the strong inverse relationship between age and CAG in the Huntington's disease cohort **(B)**. **D)** As a control we performed the association analysis between age and EEG activity for the age-matched HC group as well and observed that the association between age and the EEG activity was markedly reduced (Pearson's $r = –0.01$, Spearman's $\rho = –0.02$, $P > 0.05$) (**Supplementary Figure 1D**). Taken together, these data are broadly consistent with the view that the EEG differences could reflect Huntington's disease-specific downstream consequences of the number of CAG repeats in patients. In all panels, r depicts the Pearson's correlation coefficient while ρ depicts the Spearman-rank correlation coefficient. Abbreviations: θ/α = theta-alpha, CAG = cytosine adenine guanine, CAP = CAG-age product, EEG = electroencephalographic, FPR = false positive rate, HC = healthy controls, HD = Huntington's disease, *HTT* = huntingtin gene, LDA = linear discriminant analysis, *mHTT* = mutant *HTT*, ROC = receiver operator characteristic, TPR = true positive rate.

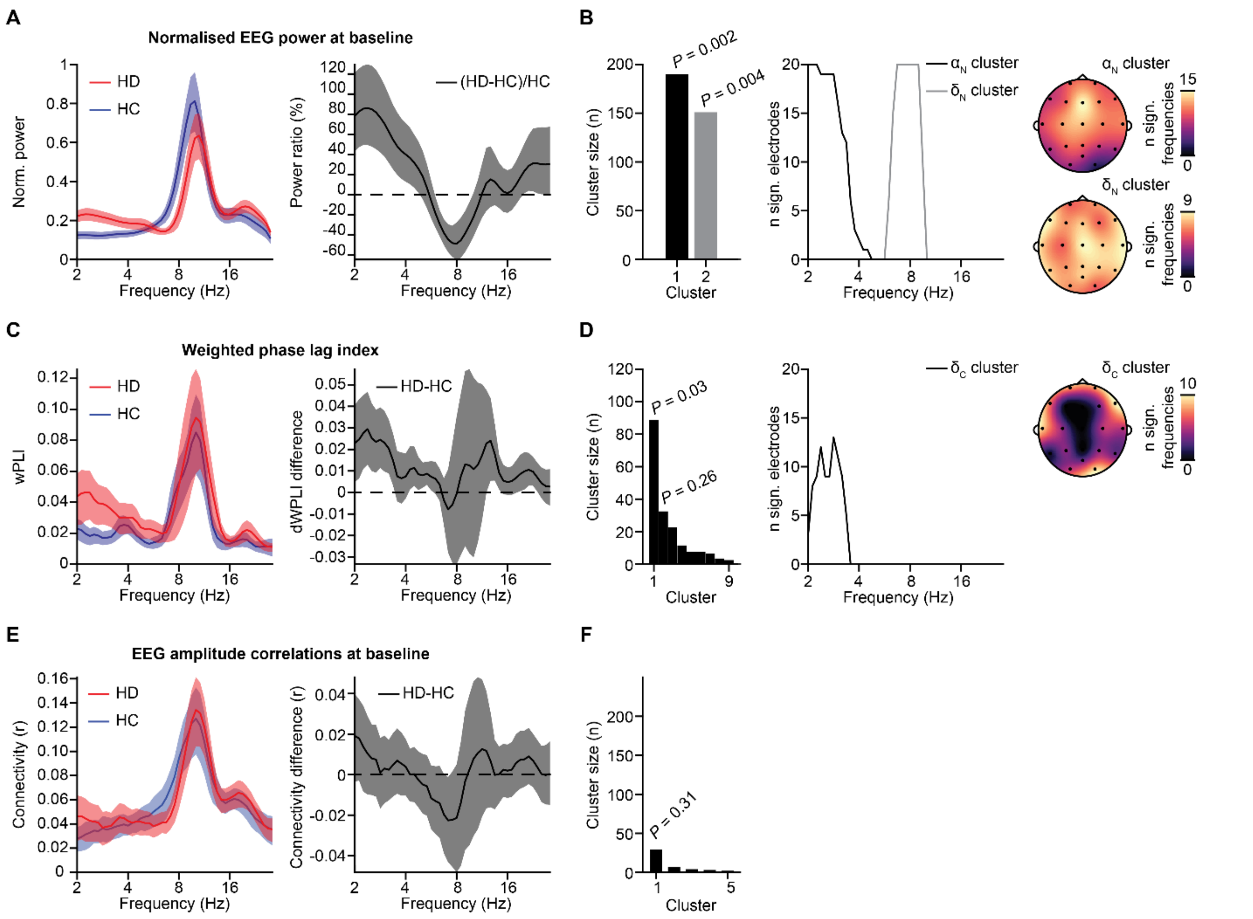

Supplementary Figure 2 Detailed EEG phenotype of early-manifest Huntington's disease – relative power and connectivity. A) Same as **Fig. 1B)** for power spectra that were normalized for each patient and control subject. **B)** Same as **Fig. 2A)** for normalized EEG activity. **C–D)** Same as **A–B)** for global connectivity among all EEG electrodes as measured with the weighted phase lag index. **E–F)** Same as **A–B)** for global connectivity among all EEG electrodes as measured with orthogonalized power correlations. Abbreviations: α_N = normalized alpha, δ_N = normalized delta, δ_C = connectivity delta, θ/α = theta-alpha, EEG = electroencephalographic, FPR = false positive rate, HC = healthy controls, HD = Huntington's disease, ROC = receiver operator characteristic, TPR = true positive rate, wPLI = weighted phase lag index.

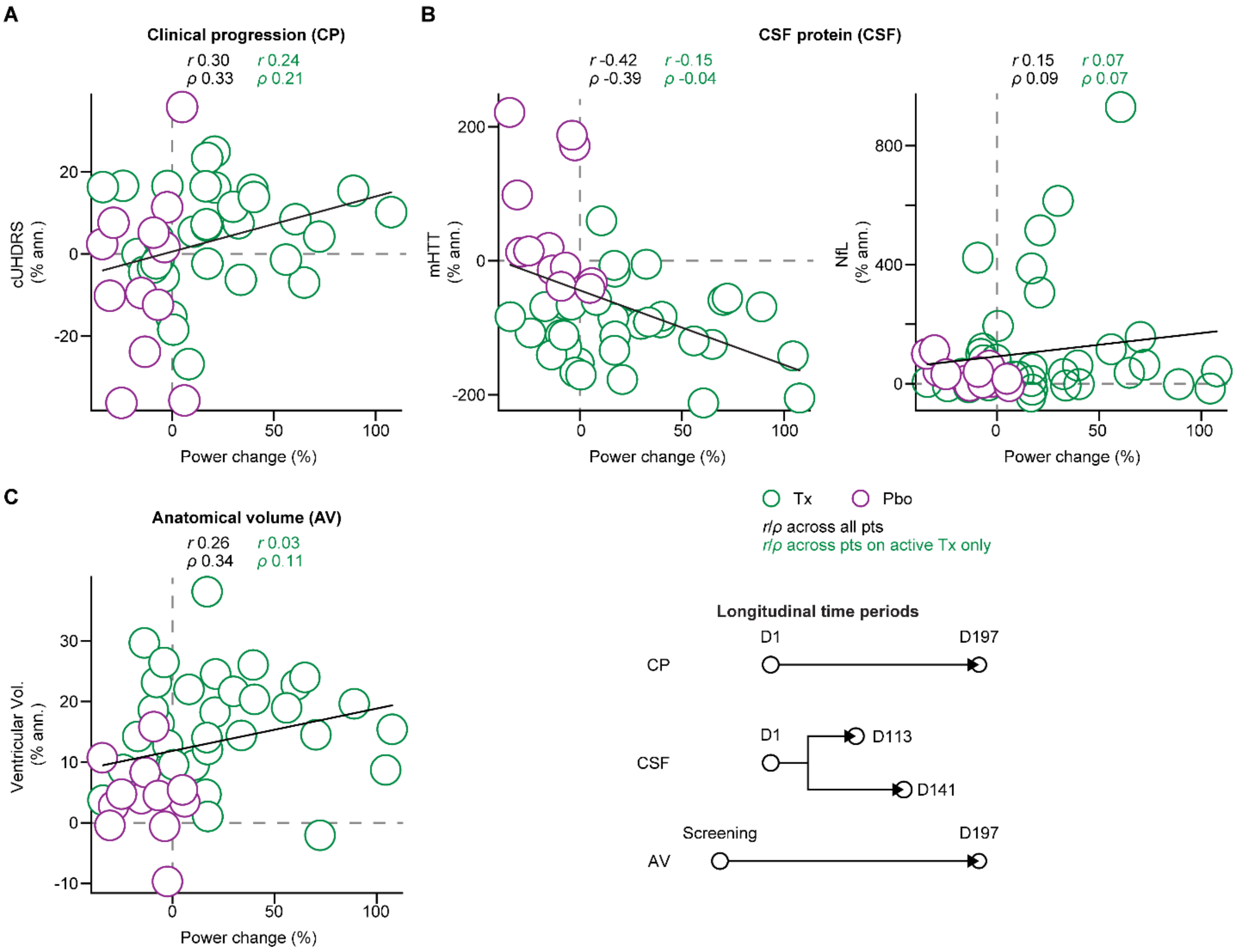

Supplementary Figure 3 Longitudinal associations of the tominersen mediated change in EEG activity. In all panels r depicts the Pearson's correlation coefficient while ρ depicts the Spearman rank correlation coefficient with green values reflecting the coefficients when limiting the analysis to the patient cohort on active tominersen treatment. **A)** Scatter plot of the change from baseline in EEG activity with the change in cUHDRS between baseline and Day 197 visits. The cUHDRS change is expressed as annualized percent change consistent with all other metrics. **B)** Same as **A)** but for the changes in CSF protein concentrations from baseline to Days 113 or 141, depending on the last CSF sampling visit of the patients. Half of the patients had their last sampling on Day 113, the other half on day 141, the annualized percent change in protein was pooled across these two sets of patients for this analysis. **C)** Same as **A)** but for the change in

ventricular volume between the screening and Day 197 visits that included magnetic resonance imaging measurements. The longitudinal volumetric change was measured with the BSI and is expressed as annualized percent change. As a convention for the imaging data, positive BSI change reflects atrophic change with an enlargement of the lateral ventricles. Abbreviations: AV = anatomical value, BSI = boundary shift integral, CP = clinical progression, CSF = cerebrospinal fluid, cUHDRS = composite Unified Huntington's Disease Ratings Scale, mHTT = mutant huntingtin protein, NfL = neurofilament light protein, Pbo = placebo, Tx = treatment.

References (supplementary materials)

1. Hipp JF, Hawellek DJ, Corbetta M, Siegel M, Engel AK. Large-scale cortical correlationstructure of spontaneous oscillatory activity. *Nat Neurosci*. Jun 2012;15(6):884-90.
2. Hawellek DJ, Schepers IM, Roeder B, Engel AK, Siegel M, Hipp JF. Altered intrinsic neuronal interactions in the visual cortex of the blind. *J Neurosci*. Oct 23 2013;33(43):17072-80.
3. Vinck M, Oostenveld R, van Wingerden M, Battaglia F, Pennartz CM. An improved index of phase-synchronization for electrophysiological data in the presence of volume-conduction, noise and sample-size bias. *Neuroimage*. Apr 15 2011;55(4):1548-65.
